# Modeling *Posidonia oceanica* shoot density and rhizome primary production

**DOI:** 10.1038/s41598-020-73722-9

**Published:** 2020-10-12

**Authors:** Elena Catucci, Michele Scardi

**Affiliations:** 1 Department of Biology, University of Rome “Tor Vergata”, via della Ricerca Scientifica, 00133 Rome, Italy; 2 CoNISMa, Piazzale Flaminio, 9, 00196 Rome, Italy

**Keywords:** Ecological modelling, Ecology

## Abstract

*Posidonia oceanica* meadows rank among the most important and most productive ecosystems in the Mediterranean basin, due to their ecological role and to the goods and services they provide. Estimations of crucial ecological process such as meadows productivity could play a major role in an environmental management perspective and in the assessment of *P. oceanica* ecosystem services. In this study, a Machine Learning approach, i.e. Random Forest, was aimed at modeling *P. oceanica* shoot density and rhizome primary production using as predictive variables only environmental factors retrieved from indirect measurements, such as maps. Our predictive models showed a good level of accuracy in modeling both shoot density and rhizome productivity (R^2^ = 0.761 and R^2^ = 0.736, respectively). Furthermore, as shoot density is an essential parameter in the estimation of *P. oceanica* productivity, we proposed a cascaded approach aimed at estimating the latter using predicted values of shoot density rather than observed measurements. In spite of the complexity of the problem, the cascaded Random Forest performed quite well (R^2^ = 0.637). While direct measurements will always play a fundamental role, our estimates could support large scale assessment of the expected condition of *P. oceanica* meadows, providing valuable information about the way this crucial ecosystem works.

## Introduction

*Posidonia oceanica* (L.) Delile, 1813, is the most widespread endemic seagrass of the Mediterranean Sea, in which its meadows represent a paramount ecosystem^1^. *P. oceanica* meadows are indeed the most valuable ecosystems in terms of goods and services they provide, and regarding their ecological role in influencing the marine coastal waters over the whole basin^2^. Besides, the primary production of *P. oceanica* meadows ranks among the largest on Earth in terms of quantity per unit surface area^2^.

In this context, estimation of *P. oceanica* productivity could result essential in an environmental management perspective and for the assessment of the ecosystem services this species provides. Clearly, direct measurements of a complex ecological process such as *P. oceanica* primary production are not only difficult to carry out, but also expensive and time-consuming, underlying the necessity for indirect methods.

In this framework, lepidochronological analysis has proven effective to tackle issues such as estimation of *P. oceanica* primary production^3–5^. The lepidochronology relies on the study of the cyclic variation of the sheaths thickness that may be observed after *P. oceanica* leaves have fallen^6^. Practically, when *P. oceanica* leaves die, the blade is lost, while the sheath remains attached to the rhizome showing cyclic variation in its thickness that has a period corresponding to a lepidochronological year^6^. Both rhizome and sheath are preserved in the *matte*, the characteristic structure formed by *P. oceanica*, with negligible morphological alterations for a very long time, e.g. many centuries^7^. Accordingly, lepidochronology allows studying the most important process driving temporal and spatial dynamics of this paramount species.

The lepidochronology gained rapidly popularity as a tool for estimating *P. oceanica* primary production^8,9^ due to its straightforward applicability, especially if compared to earlier methods, such as leaf-marking and ^14^C^10,11^. As a matter of fact, in Pergent-Martini et al.^12^ the authors drew up a standardized procedure for estimating *P. oceanica* rhizome and leaf primary production as a function of two types of data obtained from lepidochronological analyses, i.e. number and length of the sheaths, and number and length of the leaves, respectively, in conjunction with shoot density. As can be noted, shoot density is a fundamental parameter for the estimation of *P. oceanica* primary production. Furthermore, it is worth noting that the lepidochronological analysis enables estimating not only the contribution of the leaves to the overall primary production of *P. oceanica*, but also the one of the rhizomes^6,12^. Despite the rhizome primary production contributes for approximatively 6–10% of the productivity of a *P. oceanica* meadow^8,12^, the possibility of estimating the former represents a paramount advantage due to the stability over time of the rhizomes in comparison to that of the leaves. In fact, while the leaves are exposed to a wide range of pressures that can alter both their length and number, the rhizomes remain mostly unaltered due to their very slow decay, and by the fact that they shall become integral components of the *matte*^5^. The latter, as previously discussed, once built can persist with imperceptible alterations in its structure for centuries^2,7^. Besides, as the capability of creating the *matte* by *P. oceanica* is unique among the Mediterranean seagrasses, estimation of rhizome primary production could result more reliable that the one of the leaves.

In this context, this study focused on modeling both *P. oceanica* shoot density and rhizome primary production in the Italian Seas using a Machine Learning approach.

It is well known that modelling ecological processes can be an arduous task, as they involve complex interactions among biotic and abiotic factors and obviously modeling *P. oceanica* is no exception. Machine Learning approaches can provide critical advantages in empirical modeling and their potentiality has been largely demonstrated in a wide range of ecological applications^13,14^.

For instance, Machine Learning methods have been applied in modeling mortality events in the coastal rocky benthic communities over large spatial scale^15^, as well as in predicting the distribution of species of conservation interest^16^.

The crucial advantage in modeling ecological systems using Machine Learning approaches is that these methods do not impose improbable assumptions, such as linearity, on the ecological interactions between input, i.e. predictive, variables and the response. In other words, Machine Learning approaches are able to handle multifaceted and high-order interactions between predictive variables and the target, rather than merely direct causality and correlations^17,18^. Additionally, these methods do not require a set of mathematical formulations that may or may not be adequate in modeling ecological processes, rather they learn the underlying ecological patterns directly from the data. As Machine Learning approaches do not require any a priori knowledge on the nature of the relationships between predictive variables and the modelled ecological process, they further allow to exploit the potentiality of a wide range of data sources, including those obtained from maps, and other related sources of information.

Accordingly, the aim of this study was twofold. On one side, we aimed at developing Machine learning-based models of *P. oceanica* shoot density and rhizome primary production using as predictive variables only environmental factors that can be retrieved by indirect measurements, such as those based on maps. On the other side, as *P. oceanica* productivity is estimated as a function of data obtained both from lepidochronological and shoot density analyses^12^, we proposed a ‘cascaded’ approach for estimating *P. oceanica* rhizome primary production using modelled, i.e. predicted, shoot density values, rather than observed ones. The purpose of the cascaded approach is to increase the applicability of the *P. oceanica* rhizome primary production model even in cases in which data on shoot density, which require laborious field activities, are not available.

## Materials and methods

### Study area and environmental variables

The data set used in this study included 192 sites in which lepidochronological data and shoot density were acquired between 1994 and 2003. Clearly, the rhizome primary production of *P. oceanica* was estimated as defined by Pergent-Martini et al.^12^.

**Figure 1:**
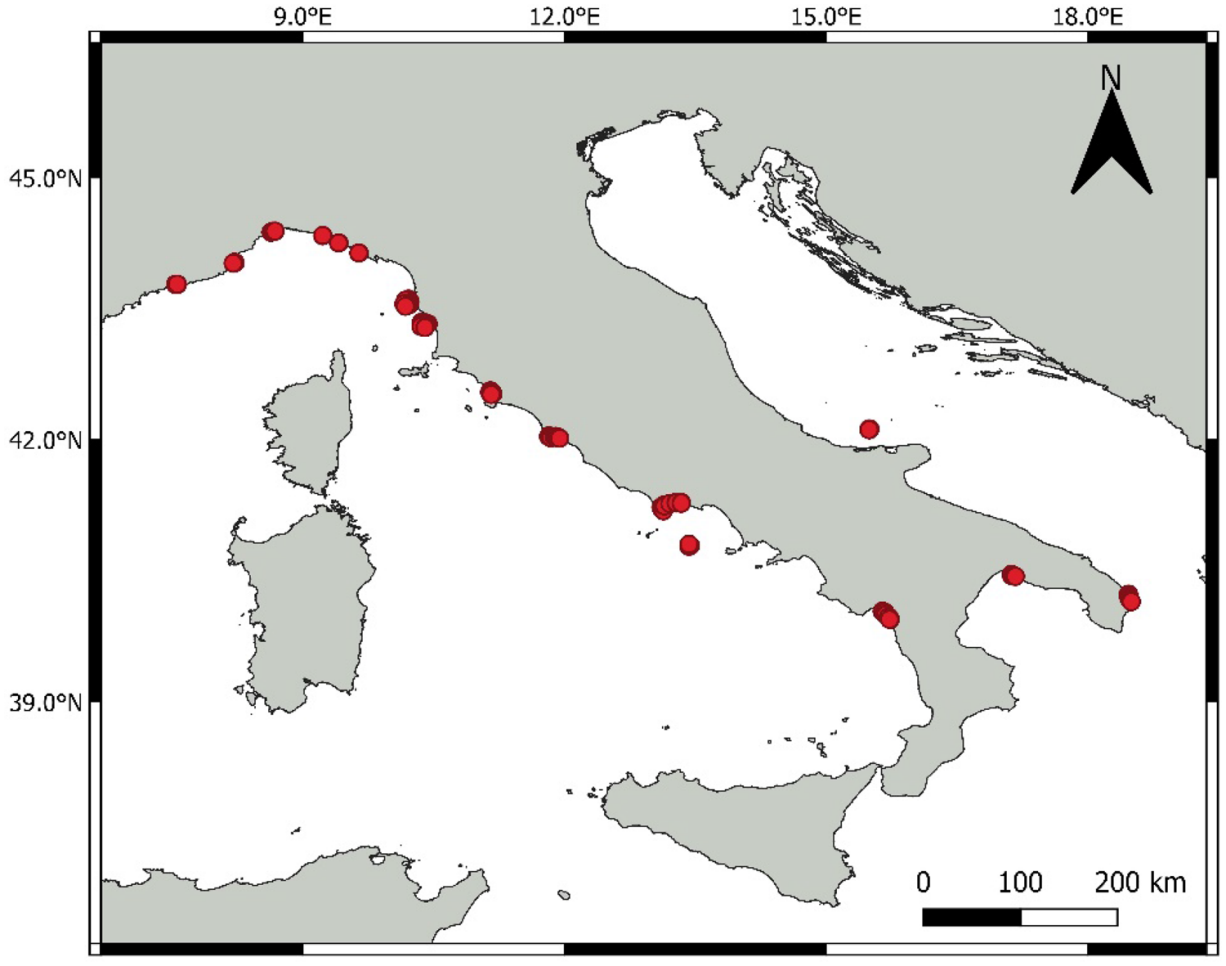
Sampling sites from which field data and indirect measurements have been collected (red circles). Data about several sampling stations are available at each site (N = 6 to 15).

The spatial coverage of the data set was not uniform across the Italian Seas. In fact, the sampling sites were mainly concentrated in five Italian regions, i.e. Liguria, Tuscany, Lazio, Basilicata and Apulia (Fig. 1).

The environmental variables were all acquired from maps and other related information sources (Table 1), according to the main aim of the study. A detailed explanation of these variables and of the methodology for their acquisition is given in the supplementary materials.

**Table 1:** Environmental factors used as predictive variables for developing *P. oceanica* models.

1	Latitude
2	Longitude
3	Depth
4	Gradient
5	Agreement between gradient and angular range of prevailing winds
6	Agreement between gradient and prevailing winds
7	Profile of the isobaths: linear
8	Profile of the isobaths: convex
9	Profile of the isobaths: concave
10	Sea floor irregularity
11	Coastline openness
12	Exposure to prevailing winds
13	Type of sea floor: sand
14	Type of sea floor: rock
15	Type of sea floor: matte
16	Disturbances: anchoring
17	Disturbances: sewage
18	Disturbances: inorganic pollution

According to the main aim of the study, these predictive variables were acquired from indirect measurements, e.g. maps.

Since these environmental factors were used as predictive variables in the modeling procedure, their selection was based on the ecological nature of the modelled processes, taking into account their influence on the latter. For instance, it is well known that depth plays a crucial role in determining the properties of *P. oceanica* meadows, such as density and productivity, as it is strictly related to other fundamental environmental factors, e.g. light. Therefore, both depth and gradient were considered as predictive variables, as well as the profile of the isobaths, described as either linear, convex or concave. The presence of sources of disturbance, such as sewage discharge or similar pollution, was also taken into account, as an increase in turbidity following an excessive enrichment from nutrient inputs might entail a reduction of water transparency and light penetration, which in turn can alter the ecological proprieties of a *P. oceanica* meadow. As for the sea floor typologies, i.e. sand, rock and matte, sources of disturbance have been represented as binary variables because of the intention of using only indirect methods for data acquisition, e.g. maps. Clearly, with such types of data source it was possible to perform, with good confidence, only a qualitative assessment. A quantitative coding of those predictive variables would indeed require expensive and time-consuming efforts for field activities, leading to a major drawback of the proposed approach.

The data set was partitioned into two subsets, i.e. training and test sets, for modeling purposes. Data partitioning represents a critical step in modeling, whose aim is obtaining two subsets that are as much as possible independent from each other, while simultaneously representative of the modelled problem, in order to avoid modeling artifacts and to ensure the applicability of the resulting models^18^.

Accordingly, the partitioning was not based on random selection of the data, rather the subsets were obtained on the basis of the following approach. The data were stratified according to depth, i.e. they were sorted on the basis of their depth and assigned to one of the following bathymetric classes, i.e.[0,5] m, (5,10] m, (10,15] m, (15,20] m, (20,25] m, (25,35] m. These classes comprised 16.67%, 23.96%, 27.08%, 17.71%, 9.90% and 4.69% of the total number of records, respectively. Subsequently, within each bathymetric class, about 70% of the data, i.e. n = 136, were assigned to the training set, while the remaining ones, i.e. n = 56, to the test set. While the former subset comprising the majority of the data was used for the training procedure of the Machine Learning algorithm, i.e. Random Forest^19^, the test subset was only used a posteriori to evaluate model performance.

The rationale behind the aforementioned approach is that the depth has a paramount ecological role in regulating both *P. oceanica* shoot density and rhizome primary production, as previously noted. In fact, a wide range of environmental conditions are related to depth, such as light, water movement and sedimentation flows, which in turn strictly affected the structure, the functioning and the ecological condition of *P. oceanica* meadows. Therefore, using the abovementioned strategy in the data allocation, the inherent variability of the ecological patterns was properly distributed among the subsets, thus ensuring the possibility of obtaining ecologically sound models.

### Random Forest

The Random Forest (RF) is a Machine Learning technique which fits an ensemble of Classification Trees and combines their predictions into a single model^19^.

RF has proven effective in a wide range of applications as it is able to address, for example, both regression and classification problems^20^, to perform cluster analysis and missing values imputation^21,22^.

RF has been used for predicting current and potential future spatial distribution of plant species^23^, as well as for estimating the marine biodiversity on the basis of the sea floor hardness^24^. RF has been also applied in ecological applications as a classification tool for the assessment of the vulnerability of *P. oceanica* meadows over a large spatial scale^25^, and for land cover classification using remote sensing data^26,27^.

This method relies upon one of the main features of Machine Learning methods, namely that an ensemble of ‘weak learners’ usually outperforms a single ‘strong learner’^19^. As a matter of fact, each Classification Tree in the forest represents a weak learner, i.e. a single model, trained on a partly independent data subset, i.e. on a bootstrap sample. Each Classification Tree provides predictions based on the data contained in its bootstrap sample, and many trees are combined into an ensemble model, i.e. into a ‘forest’. The overall output of a RF is obtained by averaging the outcomes of all the trees for regression applications, while it is based on majority voting for classification problems.

The diversity of the trees in the forest is ensured by the use of random subsets of data for the tree-building process, i.e. bootstrap samples, as well as by making a random subset of predictive variables available for the tree splitting procedure. These features allow the RF to reduce the correlation among its Classification Trees, while keeping the variance relatively small, thus leading to a more robust model^19^.

The selection of a random subset of predictive variables at each split ensures maintaining a certain level of randomness during the tree construction process^28^, and is necessary for the proper functioning of RF. As a matter of fact, the size of the random subset of predictive variables available for the tree splitting procedure represents a tuning parameter, defined as *mtry*. The latter together with the minimum number of records to be contained in each leaf, called *nodesize*, are the main tuning parameters that deeply affect RF performance^21,29^.

In its original work, Breiman^19^ suggested to set the *mtry* value equal to *p*/*3* for regression applications, being *p* is the total number of predictors, and tuning it from half to twice its original value. On the other hand, *nodesize* and *ntree* (the latter parameter is the total number of Classification Trees in the forest) are more related to the generalization ability of the RF, and to the overall complexity of the model. Growing a very large forest, e.g. *ntree* > 500, or growing the trees to achieve a high degree of purity at their leaves, e.g. *nodesize* < 5, could substantially increase the computational costs, leading to an extremely complex model^21^. It has been largely demonstrated that these parameters have to be tuned considering the available data, as large data set might require larger *nodesize* and smaller *ntree* values^25,28,30–32^.

As the goal in modelling is to obtain a model showing a high level of accuracy while presenting an appropriate level of complexity, which might vary according to the nature of the modelled process without exceeding^18^, in this study the RF training, involving the calibration of the tuning parameters, was performed as follows.

The *mtry* parameter was tested in the [3, 12] range as the data set included 18 predictive variables, while the *nodesize* was tested in the [1, 10] interval of values, setting *ntree* to 1000. The moderate size of the available data set (N = 192) allowed to grow quite large forests representing trees almost grown to their maximum depth. The resulting 100 RF configurations (i.e. 10 *mtry* values, times 10 *nodesize* values, times 1 *ntree* value) were trained using only the data contained in the training set, while their performances were assessed on the basis of the withheld data, i.e. of the test set.

The abovementioned RF training was performed for developing all the predictive models, i.e. (1) the shoot density (as shoots m^−2^) model, (2) the rhizome primary production (as g DW m^-2^ y^−1^) model based on known shoot density, (3) and the cascaded rhizome primary production (as g DW m^-2^ y^−1^) model based on predicted shoot density (see “Cascaded approach for modeling P. oceanica rhizome primary production” section).

Both model training and evaluation (see “Model evaluation” section) were performed in R^33^ environment using the package *randomForest*^20^ which implements the original RF algorithm developed by Breiman^19^.

### Cascaded approach for modeling *P. oceanica* rhizome primary production

As previously noted, shoot density is one of the fundamental parameters in the estimation of rhizome primary production, basing on the standardized approach proposed by Pergent-Martin et al.^12^.

Due to the fact that data on shoot density are obtained from laborious field activities, usually expensive and time-consuming, we proposed a cascaded approach aimed at modeling the rhizome primary production of *P. oceanica* using predicted density values, rather than observed ones. In a general perspective, the use of predicted values of shoot density could zero out survey costs.

In a methodological perspective, predicted shoot density values are meant to be used at run time, thus they were used when assessing the RF performance, while observed data were only used during the training procedure. In other words, predicted values of shoot density, provided by our predictive model, were included in test set for evaluating the performance of the cascaded model of rhizome primary production, while the training procedure of the latter was instead carried out using data obtained from direct measurements, i.e. observed data on shoot density.

The rationale behind that solution is based on practical as well as methodological reasons. In fact, it has to be considered that during the training phase the RF is aimed at detecting patterns in the data, learning how the predictive variables are related to the target. On the other side, when the model is applied to the test set, its ‘learning ability’ is assessed and the way the model is meant to be applied, i.e. with no field measurements, must be taken into account.

Accordingly, the use of the observed data on *P. oceanica* shoot density in the training procedure allowed the RF to learn the underlying interactions between the predictive variables, including shoot density, and the target, i.e. rhizome primary production. On the contrary, the use of predicted data during the model evaluation allowed testing the capability of the RF in modeling the multifaceted relationships between predictive variables and target, including density-productivity ones.

### Model evaluation

As previously noted, the models’ performance was evaluated using the data included in the test set, which are those never seen by the RF during its training. The performance of each model was evaluated by computing the determination coefficient (R^2^), which measures the proportion of target variance explained by the model, and the Mean Squared Error (MSE). The final models regarding *P. oceanica* shoot density and rhizome primary production were selected on the basis of the R^2^ value, i.e. the models showing the best predictive ability (maximum R^2^ value) were selected as the final ones.

Afterwards, for developing the cascaded model of *P. oceanica* rhizome primary production, the predicted values provided by the shoot density model showing the best performance, i.e. the most accurate model, were included among the test set data of the former. The final cascaded primary production model of *P. oceanica* rhizomes was also chosen on the basis of the R^2^ value, thus selecting the RF showing the best performance.

### Relative importance of predictive variables

The assessment of the relative importance of the predictive variables is performed during the RF training on the basis of a permutation procedure. The importance of any given predictive variable is estimated on the basis of the increase in the error rate when that predictive variable is randomly permuted^19,21^. Estimation of relative importance of predictive variables is computed using the Out-Of-Bag (OOB) data, which are the records not included in the bootstrap sample for the tree-building process. These OOB data are passed down to the tree previously grown using the bootstrap sample, obtaining predictions for these OOB data. The OOB records are then passed down to the same tree once more and the values of each predictive variable, one at a time, are randomly permuted, while those of the others are left unchanged. During this second step, new predictions for the modified OOB records are obtained, which are aggregated tree by tree as the forest is constructed. Finally, the overall deviation between the estimates provided by the original and the modified OOB records is computed and regarded as a measure of the relative importance of each predictive variable^25,32^.

## Results

### Modeling *P. oceanica* shoot density and rhizome primary production

**Figure 2:**
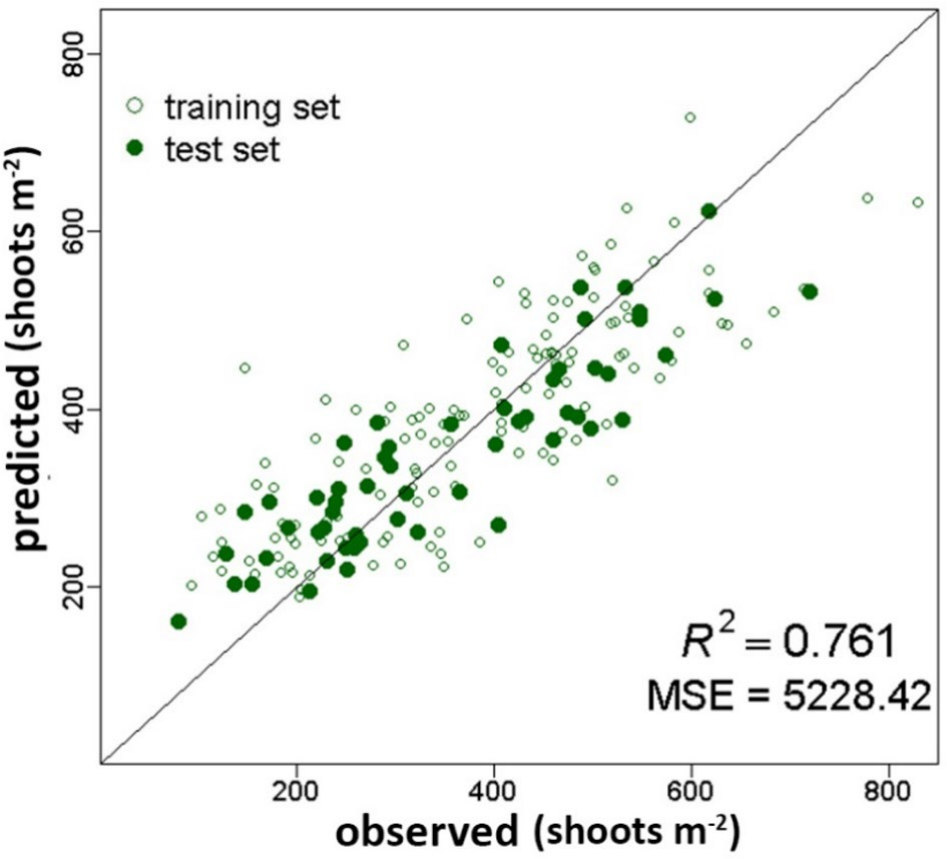
Predicted vs. observed values of *P. oceanica* shoot density (shoots m^−2^). The solid green circles showed the test set data. They are symmetrically distributed around the unit slope line and showed a good agreement between predicted and observed values. Empty circles refer to the training set data. The RF performance relative to the test set was very good i.e. R^2^ = 0.761 and MSE = 5228.42.

The RF proved effective in modeling shoot density of *P. oceanica* using environmental factors acquired only from maps as predictive variables. Indeed, the final predictive model achieved a high level of accuracy (R^2^ = 0.761), while the overall MSE was quite small, i.e. MSE = 5228.42 (Fig. 2). The square root of the latter indicated that the average error of the predictions of the test set was about 70 shoots m^−2^. The determination coefficient underlined a good agreement between predicted and observed values. In fact, as shown in Fig. 2, data were almost symmetrically distributed around the unit slope line, further pointing out that the RF output, i.e. predicted values of shoot density, was largely unbiased.

The final RF configuration of the predictive shoot density model was based on 1000 trees, almost grown to their maximum depth, i.e. *nodesize* = 2, presenting 9 randomly selected predictive variables for the splitting procedure.

**Figure 3:**
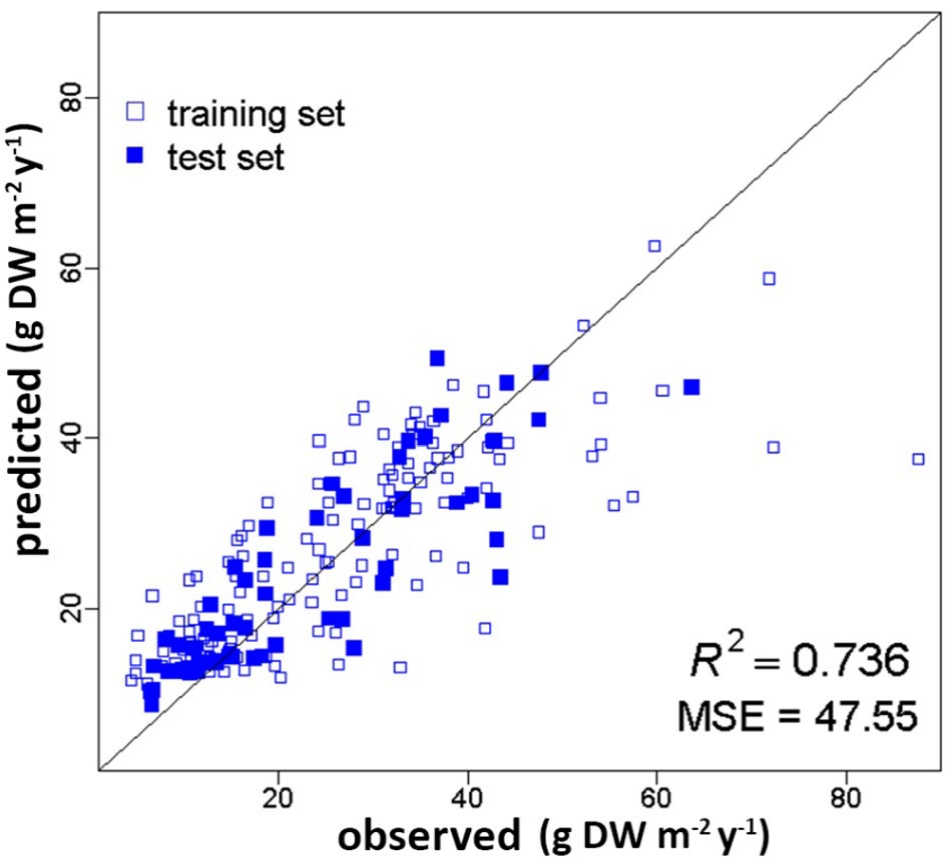
Predicted vs. observed values of *P. oceanica* rhizome primary production (g DW m^−2^ y^−1^). The solid blue squares showed the test set data. They are symmetrically distributed around the unit slope line and showed a good agreement between predicted and observed values. Empty squares refer to the training set data. The RF performance relative to the test set was very good, i.e. R^2^ = 0.736 and MSE = 47.55.

The overall performance of RF in modeling *P. oceanica* rhizome primary production was as well satisfactory (Fig. 3). In fact, its accuracy (R^2^ = 0.736) was almost as good as the one of the shoot density predictive model, showing a rather small error, i.e. MSE = 47.55 (which corresponds to an average error of the test set data around 7 g DW m^−2^ y^−1^). As can be seen in Fig. 3, the majority of the data closely matched the unit slope line, underling the good ability of the RF in modeling *P. oceanica* rhizome primary production, as expressed by the determination coefficient. These results are referred to the RF trained setting the *mtry* parameter to 7, presenting 3 cases in each leaf to stop the splitting procedure, i.e. *nodesize* = 3, and 1000 trees.

Although it might sound redundant, it worth stressing that this predictive model of *P. oceanica* rhizome primary production (Fig. 3) was built using both the 18 predictive variables together with the data on shoot density obtained from direct measurements, i.e. observed values, since it is an essential parameter in the computation of *P. oceanica* productivity^12^, as extensively discussed.

**Figure 4:**
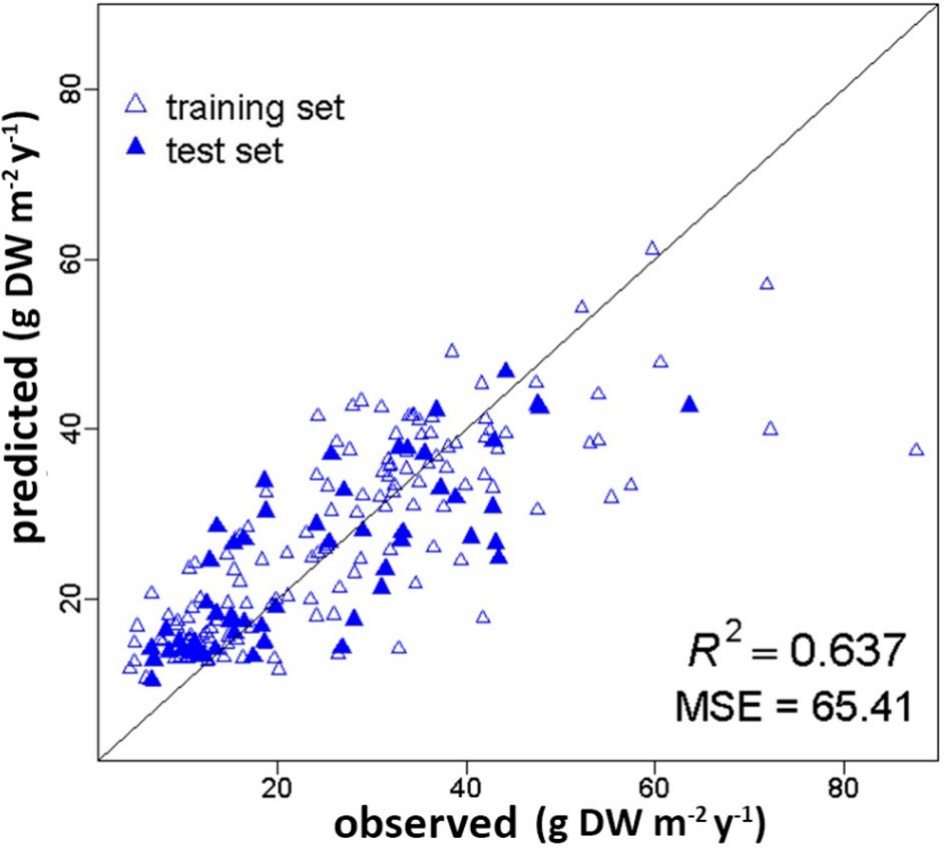
Predicted versus observed values of *P. oceanica* rhizome primary production (g DW m^−2^ y^*−*1^). The solid blue triangles showed the test set data. They are symmetrically distributed around the unit slope line and showed a good agreement between predicted and observed values. Empty triangles refer to the training set data. The RF performance relative to the test set was very good, i.e. R^2^ = 0.637 and MSE = 65.41.

The cascaded *P. oceanica* rhizome primary production model we proposed, also showed a good level of accuracy (R^2^ = 0.637). As can be seen in Fig. 4, data were once more scattered almost symmetrically around the unit slope line, indicating a good and evident agreement between predicted and observed values. The overall RF performance was quite good, with R^2^ and MSE values that were more than satisfactory, i.e. R^2^ = 0.637 and MSE = 65.41 (i.e. average error of the test set predictions around 8 g DW m^−2^ y^−1^). Despite the use of predicted shoot density data in the test set, the RF was able to explain more than 63% of the *P. oceanica* rhizome primary production variance.

The final RF configuration of the cascaded rhizome primary production model was based on 1000 trees, grown to their maximum depth, i.e. *nodesize* = 1, whose were constructed using 6 random predictive variables at each split, i.e. *mtry* = 6.

### Relative importance of predictive variables

**Figure 5:**
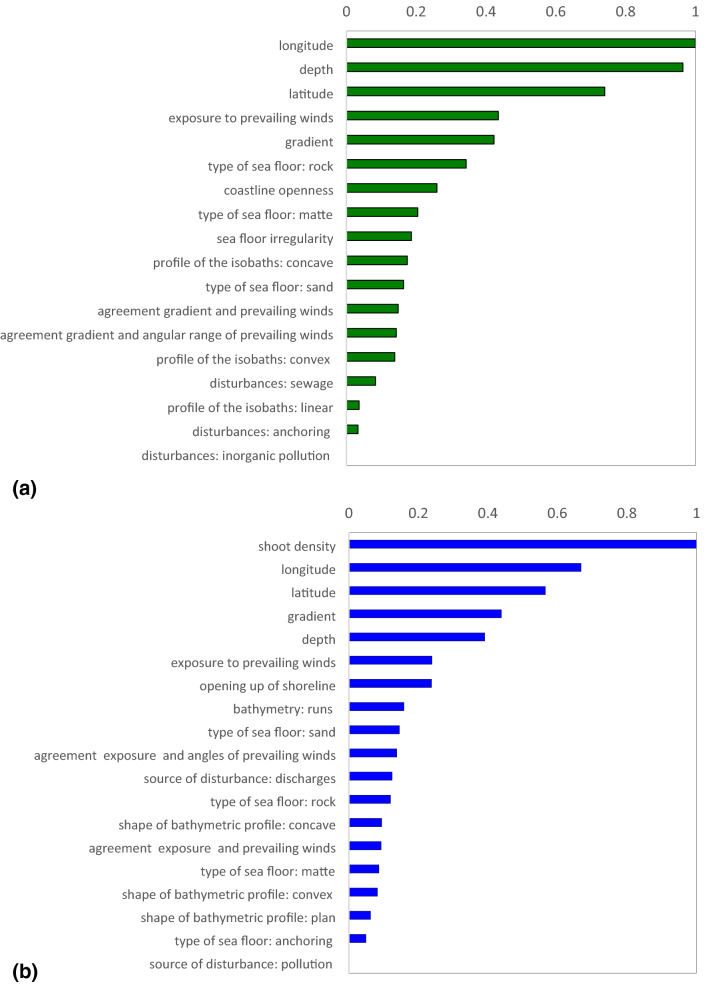

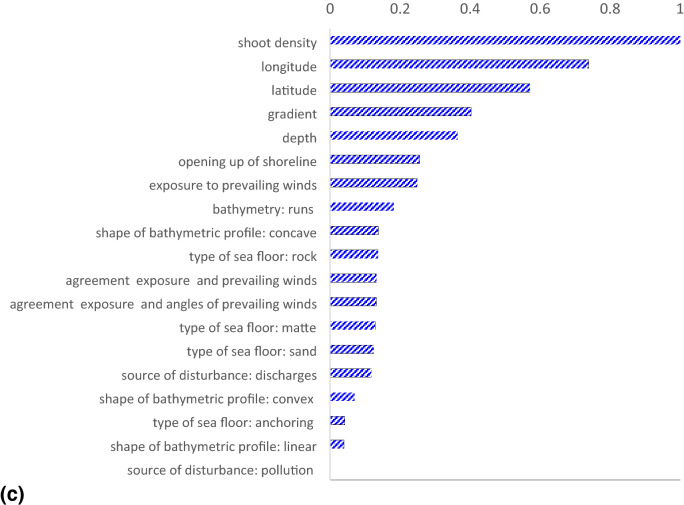
Estimates of relative importance of predictive variables assessed using the permutation measure. For each model, the results are normalized on the basis of the predictive variable showing the largest relative importance. The subplots showed: (**a**) shoot density model; (**b**) rhizome primary production model; (**c**) cascaded model of rhizome primary production.

In a RF, the relative importance of a predictive variable is quantified by the permutation measure on the basis of the error between the predictions of the original and of the modified OOB records. The estimates of the relative importance of predictive variables referring to all the three predictive models of *P. oceanica* are provided in Fig. 5.

In our results, we observed that longitude was the predictive variable showing the largest relative importance in estimating *P. oceanica* shoot density, followed by depth. The contribution of the latter was indeed as large as 96% of that provided by the former (Fig. 5a).

In a general perspective, it is worth considering that in the Italian seas while depth was certainly a more general predictive variable, in agreement with its influence on environmental factors, such as downwelling irradiance, water movement and sedimentation rates, longitude played a role in driving different predictions for meadows located in different basins. In other words, the longitude provides crucial information on the geographical location of *P. oceanica* meadows that justified its relative importance.

As a matter of fact, longitude was also the second most important predictive variable in modeling the primary production of *P. oceanica* rhizomes (Fig. 5b), and using the cascaded approach (Fig. 5c), with a contribution about as 66% and 73%, respectively, of that provided by shoot density. As expected because of its direct multiplicative role, the latter resulted the predictive variable showing the largest relative importance in both the predictive models of *P. oceanica* rhizome primary production (Fig. 5b,c).

From an ecological perspective, as well as from a purely computational viewpoint, it is obvious that *P. oceanica* productivity and shoot density have substantial dependence^5^, thus it was hardly surprising that the latter showed the largest relative importance in modeling the rhizome primary production, regardless of the followed approach (Fig. 5b,c).

Nevertheless, it is crucial to stress that estimates of relative importance of predictive variables provided by RFs did not necessarily reflect the role that these factors actually played in the underlying ecological processes. In other words, these results did not merely reflect simple cause-effect relationships between the predictive variables and the targets due to the ability of RF, such all the other Machine Learning approaches, in handling interactions that go beyond linear relations. This ability in handling non-linear relationships is expressed in the estimates of relative importance of the predictive variables which mostly reflect their role in guiding the tree-building process^34,35^, expressing their empirical nature.

## Discussion

The demand for reliable predictions is rapidly rising as environmental issues become a prominent concern of society^36^. As modeling approaches are effective tools for summarizing and synthetizing knowledge in forms allowing the formulation of quantitative, probabilistic or future states of the modelled entity^36,37^, they have gained a great interest in the modern ecosystem-based management context, which requires detailed information at all important ecological levels^37^.

The potentiality of ecological models is usually hindered by the limited availability of data, e.g. Scardi^38^. This is especially true when Machine Learning approaches are involved, as the modeling procedure is entirely driven by the data. In fact, the performance of any Machine learning-based model is strictly dependent on both the quality and the amount of available data, and *P. oceanica* modeling is not an exception to this rule.

Despite the limited amount of available data, in this study we developed predictive models showing a high level of accuracy, providing valuable information on the condition of *P. oceanica* meadows.

The model estimating *P. oceanica* shoot density exhibited a very good predictive ability (R^2^ = 0.761) showing a relatively narrow margin of error relative to the test set data (MSE = 5228.42, i.e. 70 shoots m^−2^) (Fig. 2). The conventional rhizome primary production model as well as the cascaded one, aimed at using predicted shoot density as one of the predictive variables, proved to be successful (Figs. 3, 4). Both models showed indeed good performances, i.e. R^2^ = 0.736 and R^2^ = 0.637, and quite small MSE values, i.e. 47.55 and 65.41, respectively. The latter indicated that the average error of the predictions regarding the test set was about 7 g DW m^−2^ y^−1^ in absolute value for the rhizome primary production model, and only slightly higher, i.e. 8 g DW m^−2^ y^−1^, for the model based on the cascaded approach.

**Figure 6:**
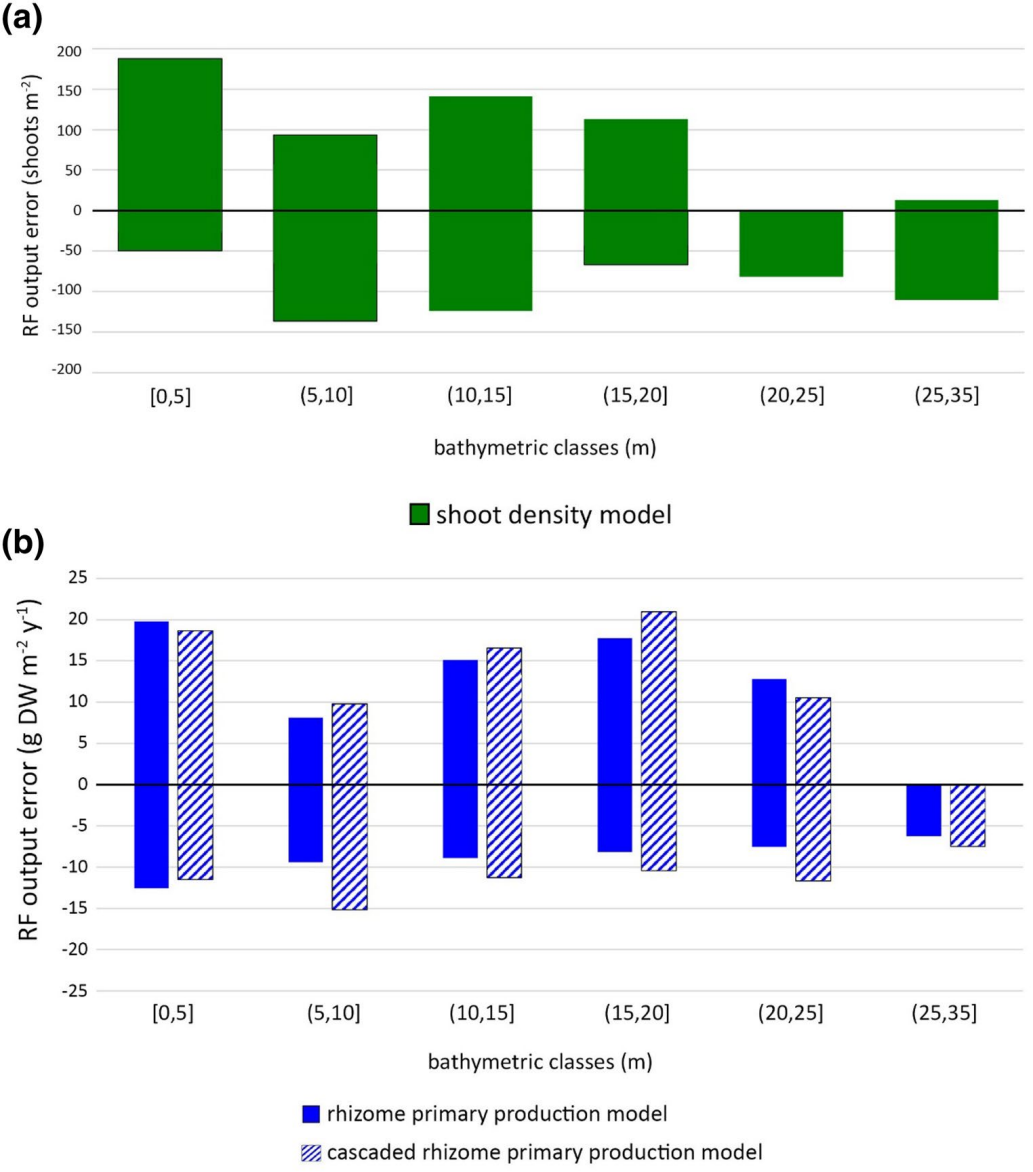
Test set error ranges with respect to six bathymetric classes: (**a**) shoot density model (green bars); (**b**) rhizome primary production model (blue bars) and cascaded approach (hatched blue bars). The bathymetric class (25,35] m included only 3 cases.

In order to fully evaluate the response of the predictive models, we analyzed the distribution of the errors of the RF outputs, regarding only the test set data, with respect to depth since the latter played a crucial role in the modeling procedure (Fig. 6). The main aim of the latter analysis was to detect whether the models showed biased results in relation to the bathymetric classes we defined.

As shown in Fig. 6a, the dispersion of the error of the RF output of the shoot density model showed a wider range for shallow waters, depth ≤ 20 m, if compared to that at deeper stands. However, regardless of the depth, the average error of the predictions of the test set data was around 70 shoots m^−2^, therefore its magnitude was still negligible if compared to density variability within *P. oceanica* meadows and to the uncertainties in the shoot counts. Accordingly, the predictive model of *P. oceanica* shoot density we developed represents a remarkable achievement, especially when considering that the latter was built on predictive variables that are not obtained from direct field activities, substantially improving its applicability.

The test set errors of both models predicting the rhizome primary production also showed a wider dispersion at depths ranging from shallow to intermediate, down to 20 m (Fig. 6b). Since in our data set the observations in that depth range, i.e.[0,20] m, represented more than 80%, these models’ behavior is possibly due to the inherent variability of *P. oceanica* meadows in that depth range. Indeed, in an ecological perspective the meadows located at shallow depth, i.e. above 20 m, are subjected to a wide range of environmental conditions, which can affect *P. oceanica* productivity and density in various manners. For instance, shallow meadows are more exposed to wave action and the resulting effects might involve substantial alterations of water turbidity and sedimentation rate, which are known to have an effect on *P. oceanica* condition.

Although water column properties play an important role, they were not taken into account for the modeling procedure because their acquisition would require direct measurements. While the latter might provide useful information, they are expensive, time-consuming and usually difficult to carry out. A critical drawback of models requiring information from field data would be that they could only be used when such data are available, thus substantially reducing their applicability. For the aforementioned reasons, we aimed at using only predictive variables that can be retrieved or inferred from maps, and possibly, in some cases, by prospecting the sampling sites when variables that are easier and inexpensive to obtain are involved.

Regarding the rhizome primary production model obtained using the cascaded approach, it can be noted that it showed a very similar distribution in relation to depth, although slightly wider, if compared to the rhizome primary production model built using direct measurement of shoot density (Fig. 6b). Obviously, the wider error distribution of the cascaded model is consistent with the slight decrease in the overall model performance.

**Figure 7:**
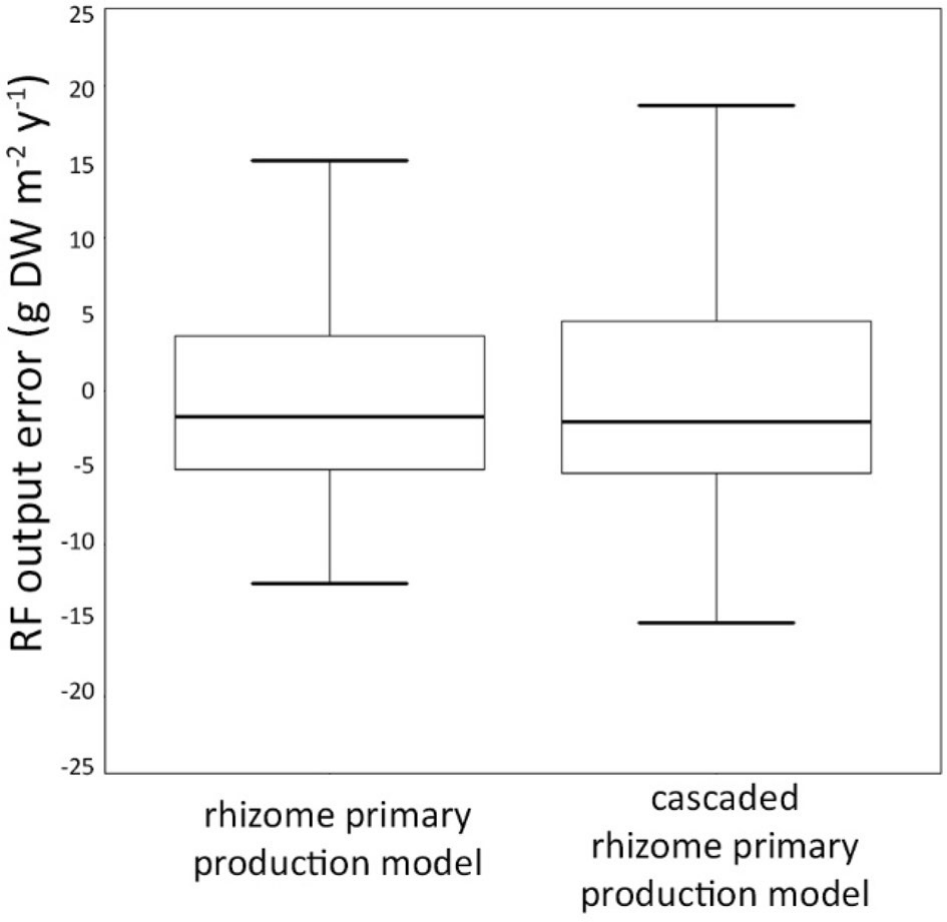
Distribution of the errors of the RF outputs for the two models of *P. oceanica* rhizome primary production, i.e. the one obtained using observed data on shoot density, and the one based on the cascaded approach. The two distributions did not differ from each other according to the Kolmogorov–Smirnov test (*p* = 0.97).

In order to compare the two *P. oceanica* rhizome primary production models, the overall errors distributions of the RF outputs of the test set were compared (Fig. 7).

As can be seen in Fig. 7, these models exhibited a very similar distribution of the RF output errors, indicating that they provided very closely matching predictions. The latter tendency was analyzed using the Kolmogorov–Smirnov (KS) test based on the null hypothesis of equal distributions of the errors of the test set data between the two rhizome primary production models. The KS test provided a value of maximum distance between the two distributions equal to 0.08, while the related *p* value was 0.97. Hence, no difference in the error distributions of the RF outputs from the two rhizome primary production models was detected. The latter underlined that the model for *P. oceanica* rhizome primary production developed using the cascaded approach provided estimates that are consistent with the ones provided by the model based on observed shoot density data.

These results pointed out the possibility of using our cascaded approach as an effective alternate solution for estimating *P. oceanica* rhizome primary production. The average values of the errors regarding the test set data of the rhizome primary production model and the one obtained using the cascaded approach, i.e. − 0.33 and − 0.37 (g DW m^−2^ y^−1^), respectively, were negligible relative to the inherent variability in our data set [4.37,87.63].

Therefore, in a general perspective, the estimates provided by our models regarding both shoot density and rhizome primary production, and similarly the ones provided by the cascaded approach which can be obtained independently of field data collection, substantially reducing the survey costs, could be considered as baselines in defining the condition of *P. oceanica* meadows. These baselines could be a valuable source of information in an environmental management perspective and for the assessment of the ecosystem services that *P. oceanica* provides.

In conclusion, we would like to stress that our predictive models on *P. oceanica* are available in the supplementary materials, together with all the information needed to reproduce our Machine Learning approaches. We believed that ecological applications of Machine Learning have not to comply with the need for the most extremely accurate predictions, rather they have to provide ecologically sound results^38^. In this context, our predictive models, which to our knowledge represent the first efforts in modeling *P. oceanica* shoot density and rhizome primary production using a Machine Learning approach, will help enhancing our understanding, with a clear ecological basis, on how *P. oceanica* ecosystems work.

## Conclusions

*P. oceanica* meadows are one of the most productive ecosystems on Earth, and they play a crucial role in controlling the sedimentation flows, in mitigating the hydrodynamic stress and in protecting the shoreline from erosion^2^. Basically, *P. oceanica* plays crucial role in the ecological balance of marine coastal waters over the whole Mediterranean basin.

In this study, we developed Machine learning-based models aimed at estimating the shoot density and the rhizome primary production using as predictive variables only the environmental factors that can be retrieved from maps in order to enhance the applicability of the resulting models, independently of field data collection.

We further proposed a cascaded approach aimed at estimating the rhizome primary production using predicted data on shoot density, rather than requiring observed measurement of the latter property, which require expensive and time-consuming field activities. The RFs showed high level of performance in modeling *P. oceanica*, allowing to obtain estimates that could prove effective in the definition of the ecological condition of meadows. Indeed, our predictive models, which to our knowledge represent the first Machine learning efforts to these purposee, provided valuable information on *P. oceanica*, enhancing our understanding on how this ecological system works.

## Supplementary information

Supplementary Information.
