# Supplementary material for: Modeling Posidonia oceanica shoot density and rhizome primary production
Source: Sci Rep. 2020 Oct 12;10:16978. doi: 10.1038/s41598-020-73722-9 (PMC7550612; doi:10.1038/s41598-020-73722-9)
Supplement: Supplementary file 1 — Supplementary Information. [file 41598_2020_73722_MOESM1_ESM.zip › supplementary_materials/predictive_variables.docx]

**Modeling *Posidonia oceanica* shoot density and rhizome primary production**

Elena Catucci^*^, Michele Scardi

| ***Predictive variables*** | |
| --- | --- |
| *1* | *latitude* |
| *2* | *longitude* |
| *3* | *depth* |
| *4* | *gradient* |
| *5* | *agreement between gradient and angular range of prevailing winds* |
| *6* | *agreement between gradient and prevailing winds* |
| *7* | *profile of the isobaths: linear* |
| *8* | *profile of the isobaths: convex* |
| *9* | *profile of the isobaths: concave* |
| *10* | *sea floor irregularity* |
| *11* | *coastline openness* |
| *12* | *exposure to prevailing winds* |
| *13* | *type of sea floor: sand* |
| *14* | *type of sea floor: rock* |
| *15* | *type of sea floor: matte* |
| *16* | *disturbances: anchoring* |
| *17* | *disturbances: sewage* |
| *18* | *disturbances: inorganic pollution* |

1. **Latitude**:

As decimal degrees, e.g. 44.37 N

1. **Longitude**:

As decimal degrees, e.g. 8.63 E

1. **Depth**:

In meters, e.g. 4.5 m

1. **Gradient**:

Find the shortest linear path passing through the point to be considered and connecting the two isobaths between which that point is located. The gradient is defined as the ratio between the depth difference between the two isobaths and the length of that path.

1. **Agreement between gradient and angular range of prevailing winds (0/1):**

If the aspect of the sea floor matches the angle from which the prevailing winds blow, then assign 1 to this variable. Otherwise, assign 0.

1. **Agreement between gradient and prevailing winds ([0.0,1.0]):**

If the aspect of the sea floor matches the angle from which the prevailing winds blow, then assign to this variable the frequency of those winds. Otherwise, assign 0.

**7 to 9. Profile of the isobaths (i.e. linear, convex or concave, respectively):**

As three mutually exclusive binary variables. Draw a circle centered at the point to be considered, setting its diameter to one nautical mile. If any one of its diameters intersects twice the isobath above that point, then set to one the binary variable for “convex”. On the contrary, if it intersects twice the isobath below that point, then set to one the binary variable for “concave”. Otherwise, set to one the binary variable for “linear”.

1. **Sea floor irregularity**

From the point to be considered, draw eight lines ¼ of nautical miles long that bisect the octants of the wind rose. If the endpoint of one of those lines lays between the same isobaths that include the center point, then assign 0 to that endpoint. Assign 1 if the endpoint is above the upper of the two isobaths that include the center point or -1 if the endpoint is below the lower isobath. Then count how many times the values assigned to the endpoints change throughout the eight octants.

1. **Coastline openness**

Number of octants in the wind rose fully opened to the prevailing winds.

1. **Exposure to prevailing winds ([0.0,1.0])**

Sum up the frequencies of the prevailing winds that blow towards the coastline throughout all the octants in the wind rose.

**13 to 15. Type of sea floor (i.e. sand, rock, matte):**

Three binary variables indicating presence or absence of sand, rock and/or matte on the sea floor. These binary variables are not mutually exclusive.

**16 to 18. Disturbances (i.e. anchoring, sewage, inorganic pollution)**

Three binary variables indicating the presence or absence of specific disturbances. These binary variables are not mutually exclusive.
